# Rotating magnetocaloric effect in highly anisotropic Tb^III^ and Dy^III^ single molecular magnets

**DOI:** 10.1038/s41598-022-20893-2

**Published:** 2022-10-05

**Authors:** Piotr Konieczny, Dominik Czernia, Takashi Kajiwara

**Affiliations:** 1 Institute of Nuclear Physics PAN, Radziwkoskiego 152, 31-342 Kraków, Poland; 2 Department of Chemistry, Faculty of Science, Nara Women’s University, Nara, 630-8001 Japan

**Keywords:** Magnetic properties and materials, Magnetic properties and materials, Ferromagnetism, Magnetic materials

## Abstract

The magnetocaloric effect (MCE) was investigated in highly anisotropic single crystals of two single molecule magnets (SMMs): [Ln^III^(Zn^II^L)_2_]CF_3_SO_3_, where Ln = Tb, Dy and L = tripodal hexadentate Schiff base ligand. The structure of these paramagnetic compounds consists of identically oriented linear trinuclear clusters in a trigonal system with an easy direction *c*∥Zn–Ln–Zn array and a hard plane *ab*⊥Zn–Ln–Zn array. The magnitude of MCE measured for *c*∥*H* was significantly greater than MCE for *ab*∥*H* at a wide temperature range regardless of the studied SMM. Therefore, the rotating magnetocaloric effect (RMCE) was evaluated. The maxima of the magnetic entropy change for RMCE were obtained at 2.0 K and moderate fields: 3.9 J K^−1^ kg^−1^ at µ_0_*H* = 1.3 T for Ln = Tb and 3.3 J K^−1^ kg^−1^ at µ_0_*H* = 1.1 T for Ln = Dy. The relative efficiency of RMCE compared to the MCE measured in *c*∥*H* was as high as 99% at low magnetic fields.

## Introduction

Magnetocaloric effect (MCE) is one of the most promising cooling technology for commercial and cryogenic applications^1^. Magnetic cooling based on MCE is considered a highly efficient and environmentally friendly alternative to the conventional gas compression method^2–4^. Although much research effort is focused on searching high efficient refrigerants near room temperature (for air conditioning or refrigerators)^5–10^, the ultra-low temperature range^11–16^ is no less important as a cost-effective alternative to ^3^He dilution refrigerators. The later application will grow in importance as a result of the development of quantum computers which require cryogenic conditions. However, most MCE refrigerants require high magnetic field changes (on the order of µ_0_Δ*H* ≈ 5–7 T), which is far above the capabilities of modern permanent magnets and therefore limits the application of MCE.

Conventional MCE is a thermodynamic process in which the magnetic material alters its temperature under the change of an external magnetic field^17–21^. However, there is another approach for magnetic cooling that involves the anisotropic magnetocaloric materials, namely the rotating magnetocaloric effect (RMCE)^22–25^. In the conventional MCE, the refrigerant is moving in and out of a magnetic field, or the external magnetic field is applied and removed. In the case of RMCE, aligned single crystals with significant magnetic anisotropy are rotated in a constant magnetic field^17,26–32^. A practical reason for this approach lies in the fact that mechanical rotations of the sample are much easier to perform and more efficient because of the operation at higher frequencies than field sweeps, thus minimizing the number of irreversible heat flows^30,33–35^. Additional energy savings for RMCE can be reached using permanent magnets.

In this work, we present the anisotropic MCE and RMCE studies on two SMM Zn^II^–Ln^III^–Zn^II^ trinuclear complexes [Ln^III^(Zn^II^L)_2_]CF_3_SO_3_, where Ln = Tb (**Tb-SMM**), Dy (**Dy-SMM**) and L denotes a tripodal hexadentate Schiff-base ligand. In our previous works ^36,37^, it was shown that both compounds are paramagnetic (no long-range magnetic order down to 2.0 K), reveal SMM behavior and have strong magnetic anisotropy with an easy axis along the crystallographic *c* axis which passes through the Zn–Ln–Zn array. The large uniaxial anisotropy makes the studied molecular magnets prospective candidates for RMCE. Single crystal magnetic measurements were performed along the easy axis (*c*∥*H*) and within the hard plane (*ab*∥*H*) to obtain the magnetic entropy change for conventional and rotating magnetocaloric effects. Both compounds reveal large RMCE at 2.0 K in moderate fields, which are easily accessible with permanent magnets.

## Results

**Figure 1:**
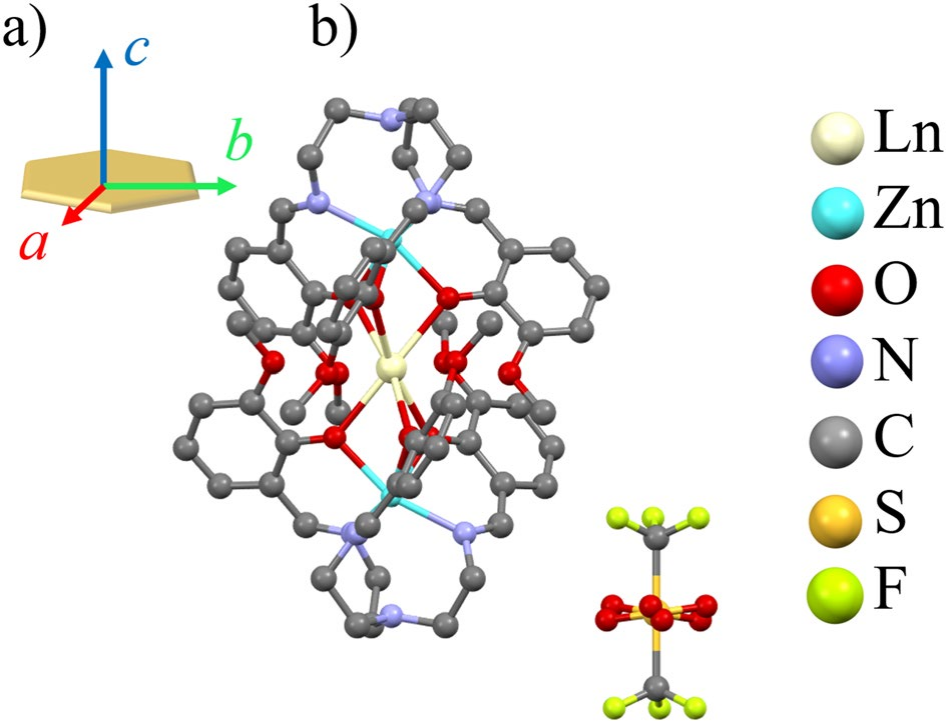
(**a**) The orientation of the monocrystal with the real space directions. (**b**) Crystal structures of the Ln^III^(Zn^II^L)_2_ unit with Ln^III^ = Tb^III^ (**Tb-SMM**), Dy^III^ (**Dy-SMM**) and the CF_3_SO_3_^−^ anion. The view is along *a* axis, and hydrogen atoms are omitted for clarity. The CF_3_SO_3_^−^ anion is disordered in two positions related by *C*_2_ axis, each with an occupancy of 0.5.

Both compounds crystallizes in the trigonal crystal system with space group *R*32, with the unit cell parameters of *a* = 11.8682(4) Å, *c* = 38.4392(16) Å, *V* = 4688.9(3) Å^3^ for **Tb-SMM** and *a* = 11.9081(4) Å, *c* = 38.2125(18) Å, *V* = 4692.7(3) Å^3^ for **Dy-SMM**, respectively^36,37^. The structure consists of two separated ions: a cationic trinuclear cluster [Ln^III^(Zn^II^L)_2_]^+^ with rare earth Ln^III^ = Tb^III^/Dy^III^ as a central ion and a non-magnetic CF_3_SO_3_^−^ anion (Fig. 1b). A crystallographic three-fold axis passes through the Zn–Tb–Zn array and is parallel to the crystallographic *c* axis. Three two-fold axes are located on Ln^III^ = Tb^III^/Dy^III^ central ion and are perpendicular to the *C*_3_ axis, and hence the molecule belongs to the *D*_3_ point group symmetry. The coordination sphere around the Ln^III^ = Tb^III^/Dy^III^ ion is fully occupied by 12 oxygen atoms, with the short-bonded phenoxo oxygen donors (2.3651(19) Å for **Tb-SMM** and 2.3437(19) Å for **Dy-SMM**) at above and below positions and long-attached methoxy oxygen donors (3.0465(15) Å and 3.076(2) Å) located at the equatorial positions. Obtained single crystals were flat and formed the hexagonal-like shape with the crystallographic axis *c* being perpendicular to the surface of the crystal and *ab* crystallographic plane lying in the plane of the surface (Fig. 1a).

**Figure 2:**
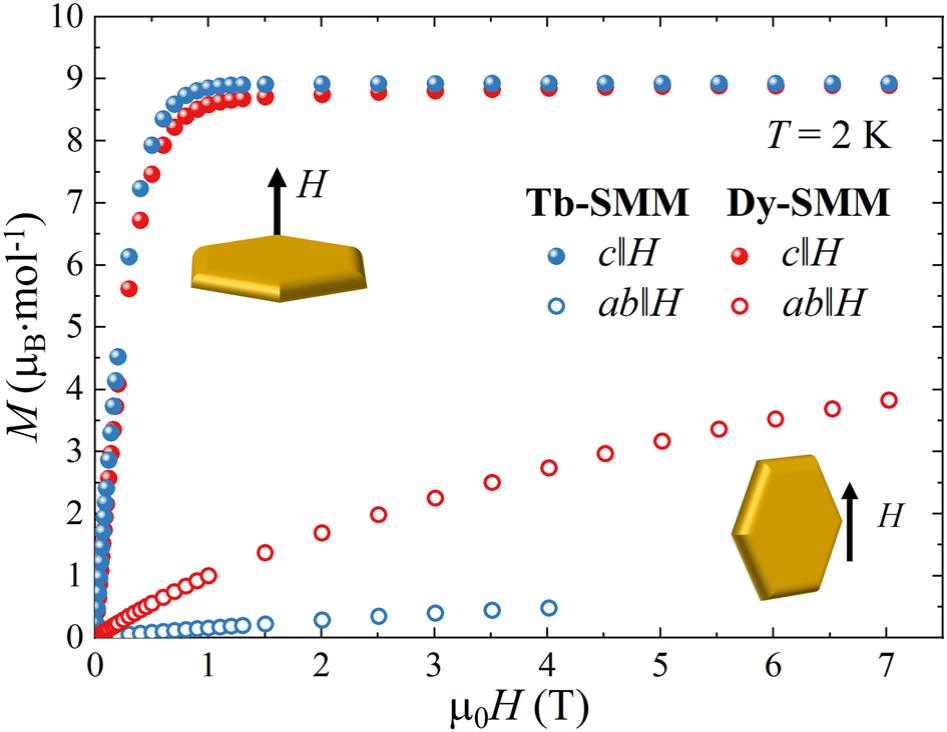
The isothermal magnetization of **Tb-SMM** and **Dy-SMM** at *T* = 2.0 K measured for *ab*∥*H* and *c*∥*H* orientations in the applied field range µ_0_*H* = 0–7 T, except for **Tb-SMM** in *ab*∥*H*, for which the highest field was µ_0_*H* = 4 T (see “Materials and methods” section). Inset pictures present the orientation of the sample regarding the external field *H*.

Single crystal magnetometry measurements of **Tb-SMM** and **Dy-SMM** were performed within the *ab* plane (*ab*∥*H*) and along the *c* axis (*c*∥*H*). Figure 2 shows the isothermal magnetization *M*(*H*) of **Tb-SMM** and **Dy-SMM** at *T* = 2.0 K. In both samples, the *c*||*H* is the easy magnetization direction, with saturation magnetization *M*_S_ ≈ 8.9 µ_B_ mol^−1^. The crystal field calculation for **Tb-SMM**^36^ revealed that in a non-zero magnetic field the lowest lying states of Tb^3+^ ions display maximal values of $\left\langle {J_{z} } \right\rangle$ =  ± 6. Taking into account *g*_Tb_ = 3/2, the expected value of saturation reaches 9 µ_B_ mol^−1^, which is close to the measured *M*_s_. In the case of **Dy-SMM** the crystal field analysis^37^ revealed that the ground state of the Dy^3+^ ion is degenerated and corresponds to the |± 13/2〉 substates, which points to ≈ 8.7 µ_B_ mol^−1^ for *g*_Dy_ = 4/3. The *ab* crystallographic plane is the hard magnetization plane with maximum values of 0.5 µ_B_ mol^−1^ (≈ 6% of *M*_S_) for **Tb-SMM** at µ_0_*H* = 4 T and 3.8 µ_B_ mol^−1^ (≈ 43% of *M*_S_) for **Dy-SMM** at µ_0_*H* = 7 T.

**Figure 3:**
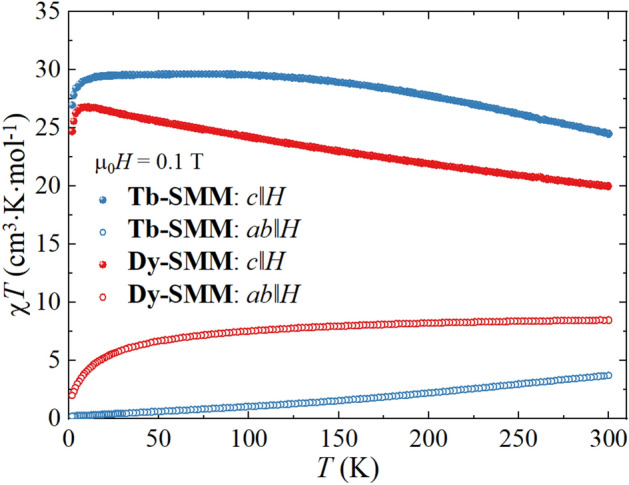
The product of molar magnetic susceptibility and temperature *χT* for **Tb-SMM** and **Dy-SMM** measured in function of temperature from 300 to 2 K for *c*∥*H* and *ab*∥*H* orientations in the static magnetic field µ_0_*H* = 0.1 T.

The dc magnetic susceptibility χ(*T*) was measured during the sample cooling from *T* = 300 K to *T* = 2.0 K in an applied magnetic field of µ_0_*H* = 0.1 T (Fig. 3). Figure 3 shows the collected data in the form of the χ*T* product for both compounds in *c*∥*H* and *ab*∥*H*. The χ*T* values for **Tb-SMM** and **Dy-SMM** reveal significant differences between the easy axis and the hard plane in the entire temperature range, which point to substantial magnetic anisotropy in both studied compounds (for detailed analysis of magnetic properties, see ^36^).

The MCE was evaluated using the indirect method for the isothermal magnetization measurements *M*(*T*, *H*) recorded in the temperature range of *T* = 2–80 K and magnetic field µ_0_*H* up to 7.0 T for *ab*∥*H* and *c*∥*H* orientations (up to 4.0 T for Tb in *ab*∥*H* orientation). The magnetic entropy change Δ*S*(*T*, *H*) was calculated using the Maxwell relationship:

1 $$\begin{array}{c}\Delta S\left(T,H\right)={\mu }_{0}\underset{0}{\overset{H}{\int }}{\left(\frac{\partial M\left(T,{H}_{1}\right)}{\partial T}\right)}_{{H}_{1}}d{H}_{1}.\end{array}$$

**Figure 4:**
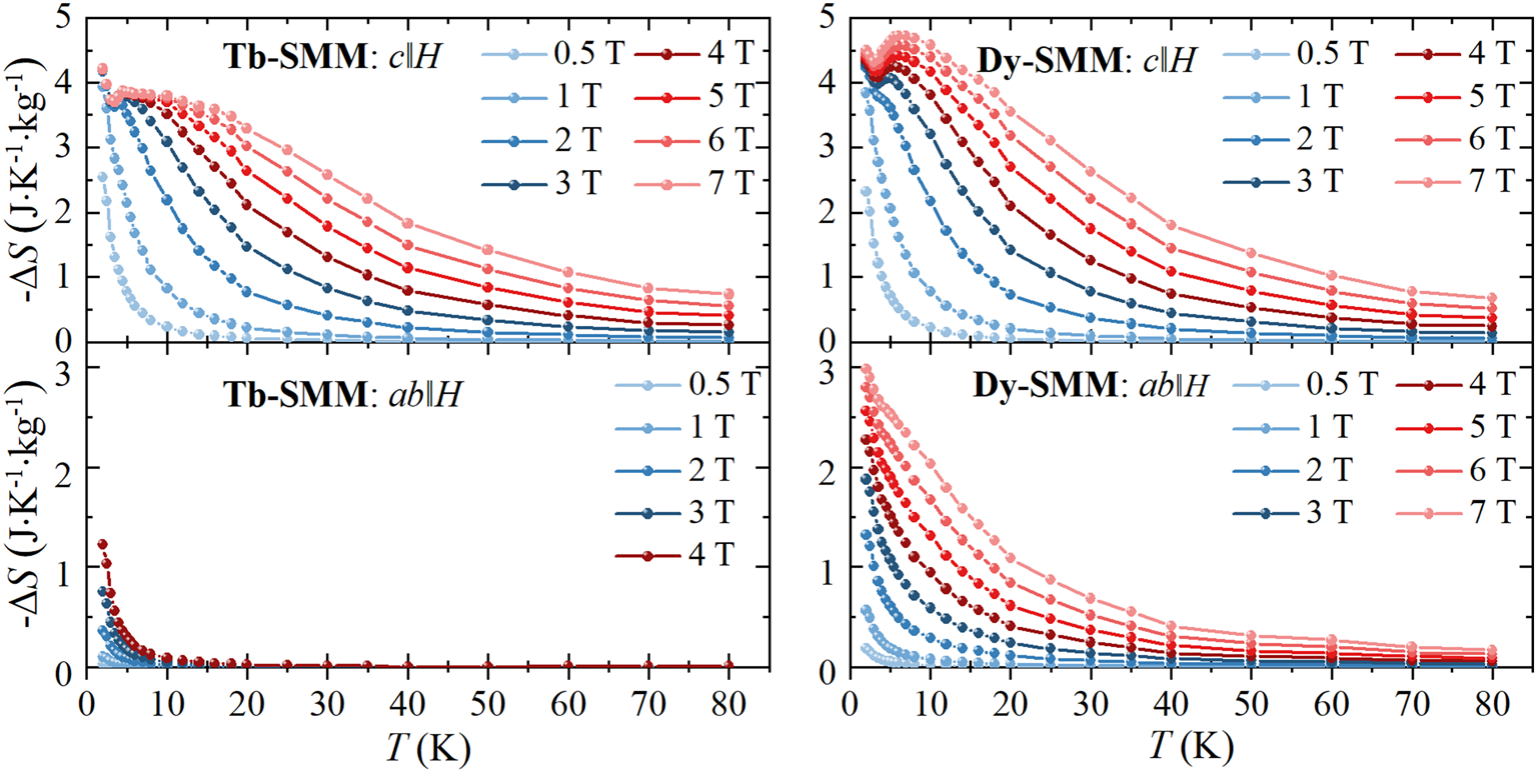
The temperature dependence of entropy change − Δ*S*(*T*, *H*) for various magnetic field changes for **Tb-SMM** and **Dy-SMM** estimated for the *c*∥*H* and *ab*∥*H* orientations. Solid lines are guides for the eyes.

The − Δ*S*(*T, H*) temperature dependence for selected fields is shown in Fig. 4. A significant difference in MCE was observed between both orientations. The magnitude of MCE was larger for *c*∥*H* than for *ab*∥*H* for both compounds, and additionally, a peak of the -*ΔS(T, H)* appeared for magnetic field µ_0_*H* ≥ 3 T in the case of *c*∥*H* but was absent for *ab*∥*H*. For **Tb-SMM**, the maximum entropy change − Δ*S*_max_ was observed at *T* = 2.0 K reaching -Δ*S*_max_ = 4.21 J K^−1^ kg^−1^ for *c*∥*H* in µ_0_Δ*H* = 7 T and − Δ*S*_max_ = 1.23 J K^−1^ kg^−1^ for *ab*∥*H* in µ_0_Δ*H* = 4 T. In case of **Dy-SMM** the maximum of − Δ*S* was found in µ_0_Δ*H* = 7 T reaching − Δ*S*_max_ = 4.72 J K^−1^ kg^−1^ at *T* = 6.0 K for *c*∥*H* and − Δ*S*_max_ = 2.98 J K^−1^ kg^−1^ at *T* = 2.0 K for *ab*∥*H.*

**Figure 5:**
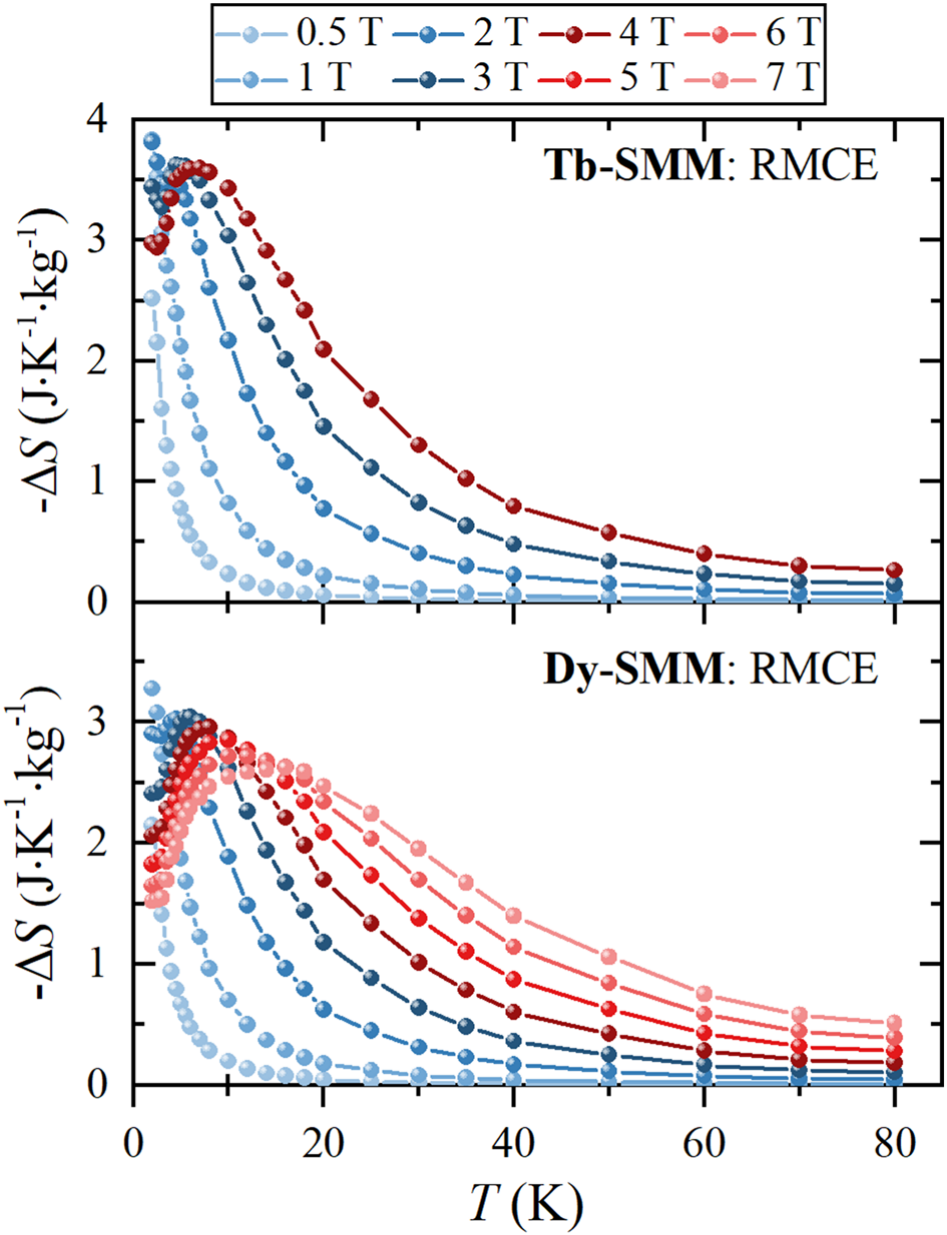
The RMCE entropy change − Δ*S*(*T*, *H*) as a function of the temperature *T* ranging from 2 to 80 K for the selected magnetic fields µ_0_*H* for **Tb-SMM** and **Dy-SMM**. Solid lines are guides for the eyes.

To study the RMCE, the magnetic entropy change related to the rotation of a single crystal was calculated as the difference − Δ*S*_R_ = − (Δ*S*_*c*∥*H*_ − Δ*S*_*ab*∥*H*_), where Δ*S*_*c*∥*H*_ and Δ*S*_*ab*∥*H*_ are the entropy changes for *c*∥*H* and *ab*∥*H* respectively. Figure 5 depicts − Δ*S*_R_ temperature dependence for **Tb-SMM** and **Dy-SMM**. One can notice the presence of the peak that was also observed for the conventional MCE for *c*∥*H*, which for the RMCE entropy change is broader. Moreover, the position of this peak has moved towards higher temperatures. The shift was from *T* = 4.5 K to *T* = 7.0 K for µ_0_*H* = 4 T for **Tb-SMM**, and for **Dy-SMM**, from *T* = 5.5 K to *T* = 8.0 K for µ_0_*H* = 4 T, and *T* = 6.0 K to *T* = 16.0 K for µ_0_*H* = 7 T.

**Figure 6:**
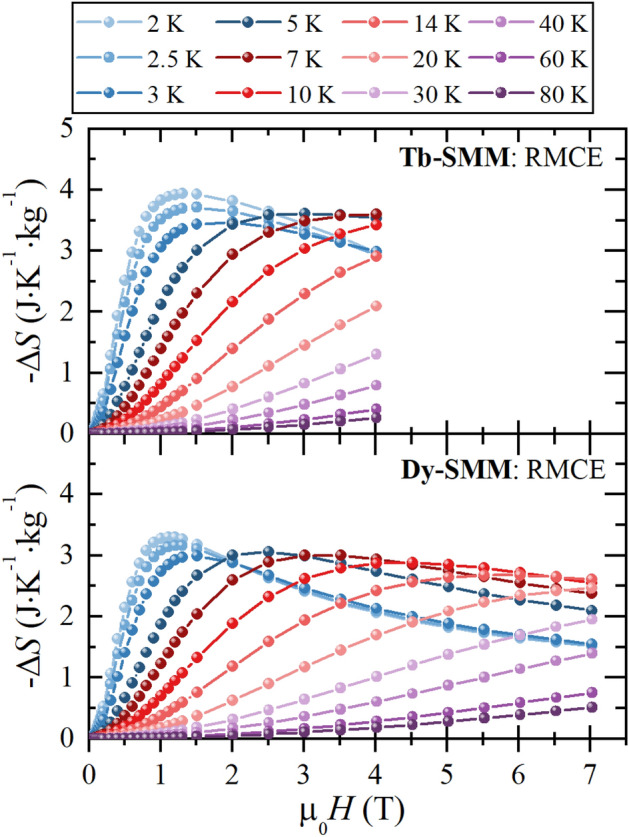
The RMCE entropy change − Δ*S*(*T*, *H*) as a function of the magnetic field µ_0_*H* up to 7 T for the selected temperatures for **Tb-SMM** and **Dy-SMM**. Solid lines are guides for the eyes.

Figure 6 shows the RMCE entropy change as a function of the applied magnetic field for selected temperatures for **Tb-SMM** and **Dy-SMM**. The maximum RMCE entropy change − Δ*S*_max_ was obtained at *T* = 2.0 K for both compounds for relatively low magnetic fields. For **Tb-SMM**, − Δ*S*_max_ was found for µ_0_*H* = 1.3 T with − Δ*S*_max_ = 3.94 J K^−1^ kg^−1^, and for **Dy-SMM** for µ_0_*H* = 1.1 T with − Δ*S*_max_ = 3.3 J K^−1^ kg^−1^. One can notice that the peak of the − Δ*S* moves towards higher temperatures with an increasing magnetic field. Therefore, for low magnetic fields, the RMCE is greater at lower temperatures, whereas high fields are more advantageous at higher temperatures. For **Tb-SMM**, in µ_0_*H* = 4 T, − Δ*S*_max_ is found at *T* = 7 K, and for **Dy-SMM** in µ_0_*H* = 7 T at *T* = 14 K.

The utility of material for magnetocaloric cooling applications can be evaluated using the proposed Temperature averaged Entropy Change (*TEC*) figure of merit ^38–41^:

2 $$\begin{array}{c}TEC\left(\Delta {T}_{\text{lift}}\right)=\frac{1}{\Delta {T}_{\text{lift}}}\underset{{T}_{\text{mid}}}{\mathrm{max}}\left\{{\int }_{{T}_{\text{mid}}-\frac{\Delta {T}_{\text{lift}}}{2}}^{{T}_{\text{mid}}+\frac{\Delta {T}_{\text{lift}}}{2}}\left|\Delta S\left(T,H\right)\right|dT\right\}.\end{array}$$

It is estimated for the specific temperature range Δ*T*_lift_ = *T*_hot_ − *T*_cold_ in which the refrigerating material can potentially work, where *T*_hot_ and *T*_cold_ are temperatures of cold and hot reservoirs, respectively. The integral is maximized for the center of the average, *T*_mid_, chosen by sweeping over the available Δ*S*(*T*, *H*) data. In our study, the temperature interval Δ*T*_lift_ was set between 1 and 30 K with a fixed step of 1 K.

**Figure 7:**
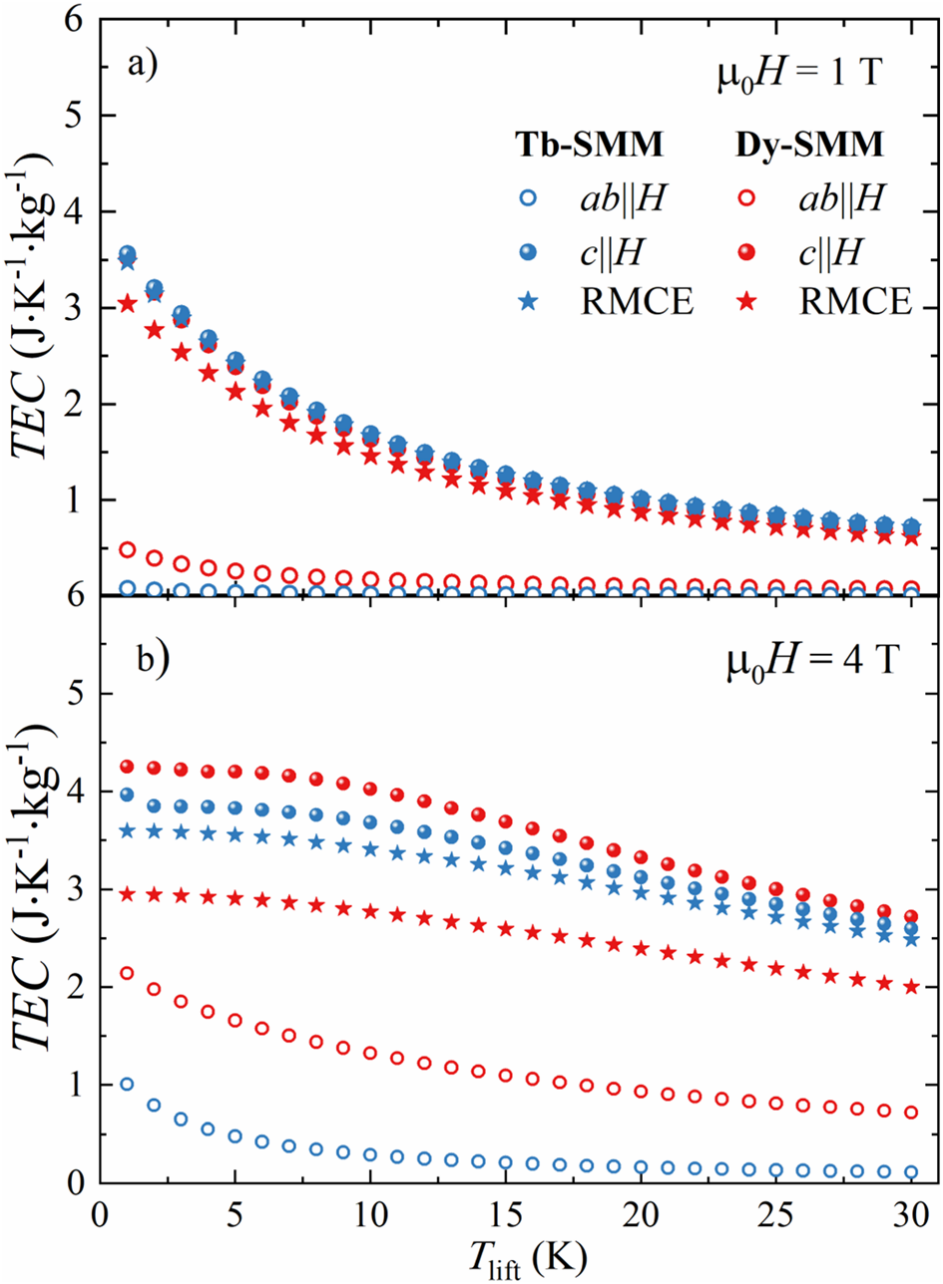
The temperature averaged entropy change (TEC) in the function of temperature interval Δ*T*_lift_ for conventional MCE and RMCE for **Tb-SMM** and **Dy-SMM** calculated for the applied magnetic field of µ_0_*H* = 1 T (**a**) and µ_0_*H* = 4 T (**b**).

The dependence of *TEC* on Δ*T*_lift_ in µ_0_*H* = 1 T, 4 T was depicted in Fig. 7 for **Tb-SMM** and **Dy-SMM** for conventional MCE in *c*∥*H* and *ab*∥*H* orientations and RMCE. As expected for *ab*∥*H*, the *TEC* was small compared to the easy axis geometry for both compounds regardless of the magnetic field value. In µ_0_*H* = 1 T, the *TEC* performances were almost identical in four cases: in *c*∥*H* orientation for **Tb-SMM** and **Dy-SMM**, RMCE for **Tb-SMM**, and slightly lower for RMCE for **Dy-SMM** (down to 85% of the corresponding *TEC* for the other three cases). The *TEC* values monotonically decreased with Δ*T*_lift_ for both compounds and all orientations in the analyzed range. The situation is different in µ_0_*H* = 4 T, for which the *TEC*(Δ*T*_lift_) curves split. Although for **Tb-SMM**, the *TEC* for RMCE still amounts to approximately 90% of corresponding *TEC* for *c*∥*H* orientation, the same ratio was reduced to 70% for **Dy-SMM**. Additionally, the *TEC* values were relatively constant in the range Δ*T*_lift_ = 1–6 K for *c*∥*H* and RMCE for both SMMs. For larger values of Δ*T*_lift_, *TEC* started to decrease linearly.

**Figure 8:**
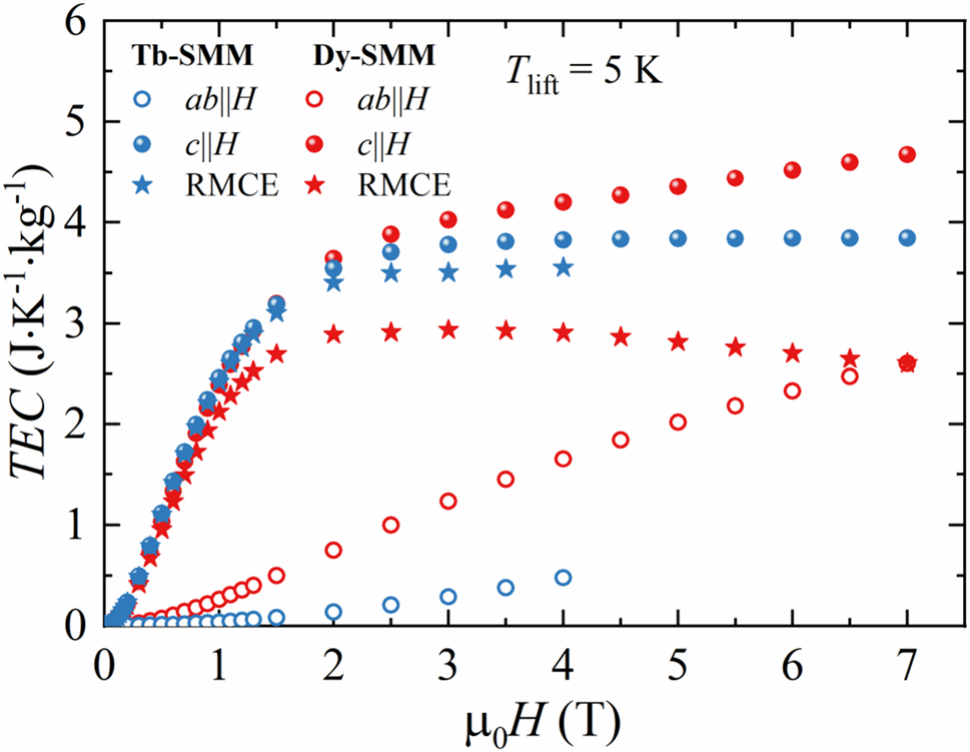
The temperature averaged entropy change (TEC) in the function of applied magnetic field µ_0_*H* calculated for temperature interval Δ*T*_lift_ = 5 K for conventional MCE and RMCE for **Tb-SMM** and **Dy-SMM**.

The temperature interval of Δ*T*_lift_ = 5 K was selected to study the field dependence of *TEC*(5) for **Tb-SMM** and **Dy-SMM**. The results are presented in Fig. 8. For **Tb-SMM** in *c*∥*H* orientation, *TEC* initially increased with the magnetic field up to µ_0_*H* = 4 T and reached a plateau for higher fields. In µ_0_*H* = 1 T, *TEC* was equal to 2.46 J K^−1^ kg^−1^, and in µ_0_*H* = 4–7 T to 3.83 J K^−1^ kg^−1^. Similar behavior was observed for RMCE for the same compound, obtaining *TEC* = 2.42 J K^−1^ kg^−1^ in µ_0_*H* = 1 T and *TEC* = 3.55 J K^−1^ kg^−1^ in µ_0_*H* = 4 T. In hard geometry, a monotonic increase of *TEC* with the magnetic field was observed with *TEC* = 0.04, 0.48 J K^−1^ kg^−1^ in µ_0_*H* = 1, 4 T, respectively. For **Dy-SMM** in *c*∥*H* orientation, *TEC* increased in the whole magnetic field range reaching *TEC* = 2.38, 4.2, 4.68 J K^−1^ kg^−1^ in µ_0_*H* = 1, 4, 7 T, respectively. The corresponding *TEC* for RMCE increased with the magnetic field up to µ_0_*H* = 3 T, and then it started decreasing. The obtained values for RMCE were equal to *TEC* = 2.13, 2.91, 2.61 J K^−1^ kg^−1^ in µ_0_*H* = 1, 4, 7 T, respectively. As for **Tb-SMM** in hard geometry, the corresponding *TEC* for **Dy-SMM** showed a monotonic increase of *TEC* with magnetic field with *TEC* = 0.26, 1.66, 2.61 J K^−1^ kg^−1^ in µ_0_*H* = 1, 4, 7 T, respectively.

## Discussion

Although the obtained − Δ*S*_max_ for **Tb-SMM** and **Dy-SMM** are approximately ten times smaller than the recently reported values for magnetic coolers based on Gd ions with − Δ*S*_max_ = 30–50 J K^−1^ kg^−1^ in µ_0_*H* = 7 T ^42–44^, it should be noted that the RMCE reported in this study brings a few essential advantages. The magnetic fields at which the RMCE exhibits − Δ*S*_max_ (µ_0_*H* = 1.3 T (**Tb-SMM**), 1.1 T (**Dy-SMM**)) are easily accessible by the permanent magnets; therefore, the potential magnetic cooler based on **Tb-SMM** or **Dy-SMM** could operate without superconducting magnets. The RMCE-based refrigerator can also work at higher frequencies and thus with greater efficiency than the conventional MCE. Last but not least, the problem of low heat conductivity and dissipation of the released heat ^21^ may be overcome due to the flat geometry of the crystals used and thus a large surface-to-volume ratio. The RMCE properties for selected compounds are compared in Table 1. In high field conditions (µ_0_*H* = 5.0 T) and *T*_max_ (the temperature at which − Δ*S* reaches maximum value), there are many examples of refrigerants revealing giant RMCE, which is much higher than those observed for **Tb-SMM** and **Dy-SMM**. However, from economical point of view, the most interesting conditions are low fields (µ_0_*H* = 1.0 T, easily accessible with permanent magnets) and *T* = 2.0 K (which can be easily reached by pumping liquid ^4^He). In these conditions (µ_0_*H* = 1.0 T and *T* = 2.0 K), both investigated compounds reveal high performance, comparable to other refrigerants with giant RMCE.

**Table 1:** Examples of rotating magnetocaloric properties of selected potential refrigerants.

Name	Refs.	− Δ*S*_max_ (J K^−1^ kg^−1^)	
		µ_0_*H* = 5.0 T and *T*_max_	µ_0_*H* = 1.0 T and *T* = 2.0 K
**Tb-SMM**	This work	3.60 (in µ_0_*H* 4.0 T) at 7.0 K	3.83
**Dy-SMM**	This work	2.85 at 10 K	3.28
Textured DyNiSi	^45^	17.6 at 13 K	≈ 3.5
TbScO_3_	^46^	23.62 at ≈ 5.0 K	≈ 3.0
TbMn_2_O_5_	^34^	13.14 at ≈ 11 K	≈ 1.8
{Dy(OAc)_3_(H_2_O)_2_}_2_] 4 H_2_O	^30^	≈ 6.0 J K^−1^ kg^−1^ at ≈ 10.0 K	≈ 4.5
Ni(en)(H_2_O)_4_SO_4_ 2H_2_O	^47^	≈ 12.0 J K^−1^ kg^−1^ at ≈ 10.0 K	≈ 0.5

Recently reported materials for conventional MCE show the variation of *TEC*(5) in µ_0_Δ*H* = 1 T between 1 and 10 J K^−1^ kg^−1^ ^41,48–53^; thus, the RMCE results reported for **Tb-SMM** and **Dy-SMM** fall in a moderate range with *TEC*(5) = 2.42 J K^−1^ kg^−1^ for the former and *TEC*(5) = 2.13 J K^−1^ kg^−1^ for the latter in the same magnetic field. The studies of the RMCE in HoNiGe_3_ single crystal ^54^ presented a much higher *TEC*(5) of approximately 12 J K^−1^ kg^−1^ in µ_0_Δ*H* = 5 T compared to the corresponding values of 3.55 J K^−1^ kg^−1^ in µ_0_*H* = 4 T for **Tb-SMM**, and 2.82 J K^−1^ kg^−1^ in µ_0_*H* = 5 T for **Dy-SMM**, but with significantly smaller entropy change in µ_0_Δ*H* = 1 T and higher temperatures for which the entropy change maximum was observed (between 5 and 15 K). Therefore, **Tb-SMM** and **Dy-SMM** are potentially more attractive candidates for ultra-low temperature cooling with permanent magnets.

**Figure 9:**
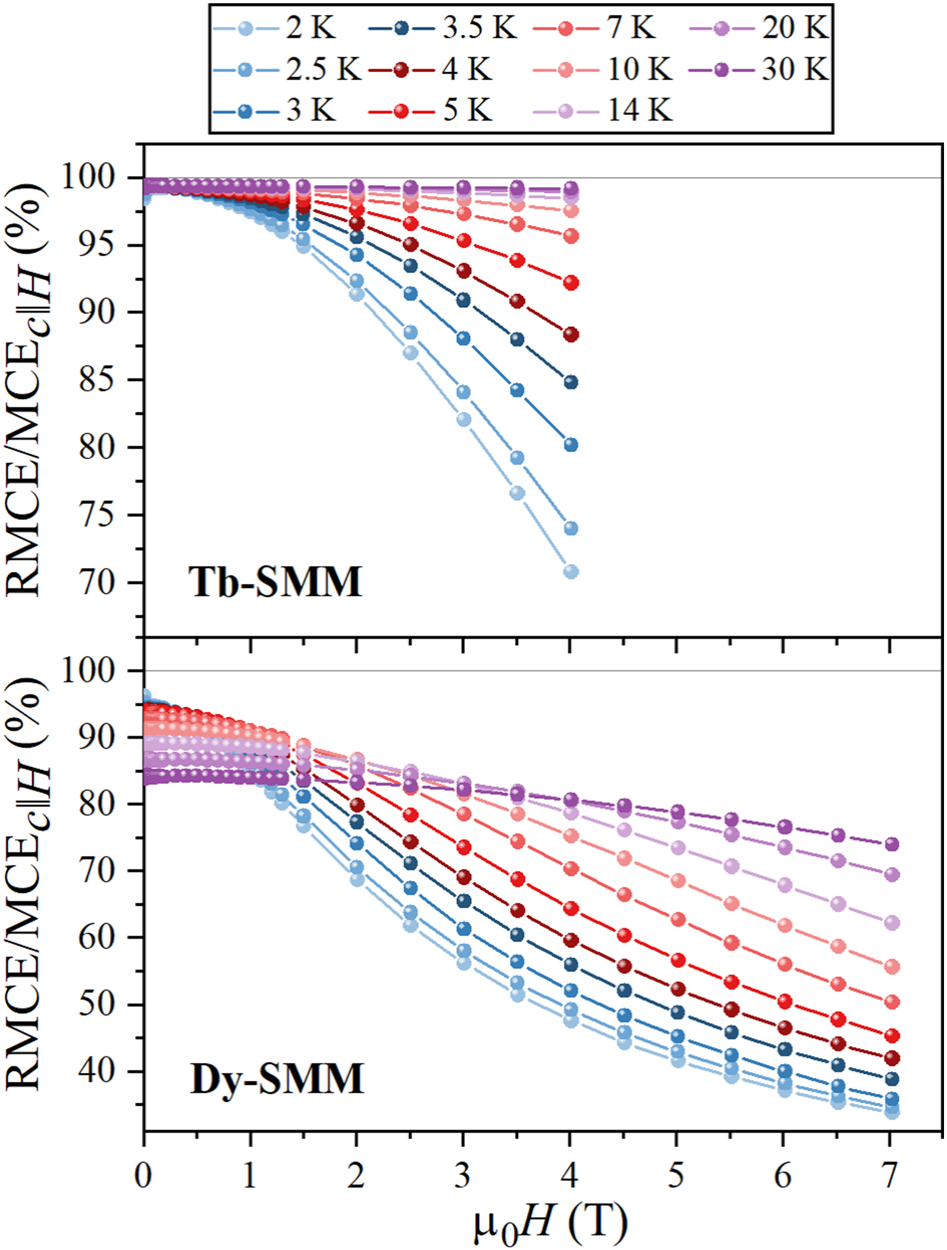
The ratio RMCE/MCE_*c*∥*H*_ between RMCE and conventional MCE measured for *c*∥*H* at *T* = 2–80 K for **Tb-SMM** in magnetic fields up to µ_0_*H* = 4 T and **Dy-SMM** in fields up to µ_0_*H* = 7 T. Solid lines are guides for the eyes.

The large difference between MCE for *c*∥*H* and *ab*∥*H* makes the RMCE nearly as efficient as the conventional MCE_*c*∥*H*_ measured for *c*∥*H*, what is pictured by the RMCE/MCE_*c*∥*H*_ ratio in Fig. 9. In the case of **Tb-SMM**, the RCME/MCE_*c*∥*H*_ value does not drop below the level of 90% for all temperatures measured in the magnetic fields up to µ_0_*H* = 2 T. Moreover, the ratio increases monotonically with the temperature in the full range of measured fields. The relative efficiency of RMCE is lower for **Dy-SMM**, for which the ratio RCME/MCE_*c*∥*H*_ was higher than 90% only for *T* = 2–10 K and magnetic fields up to about µ_0_*H* = 1 T. The difference in RCME/MCE_*c*∥*H*_ between **Tb-SMM** and **Dy-SMM** is directly related to MCE within the hard plane (*ab*∥*H*), which is substantially weaker for **Tb-SMM**. Ideally, the RMCE should be the most efficient in a system for which the conventional MCE almost vanishes in one of the crystal orientations and is large for another orientation.

**Figure 10:**
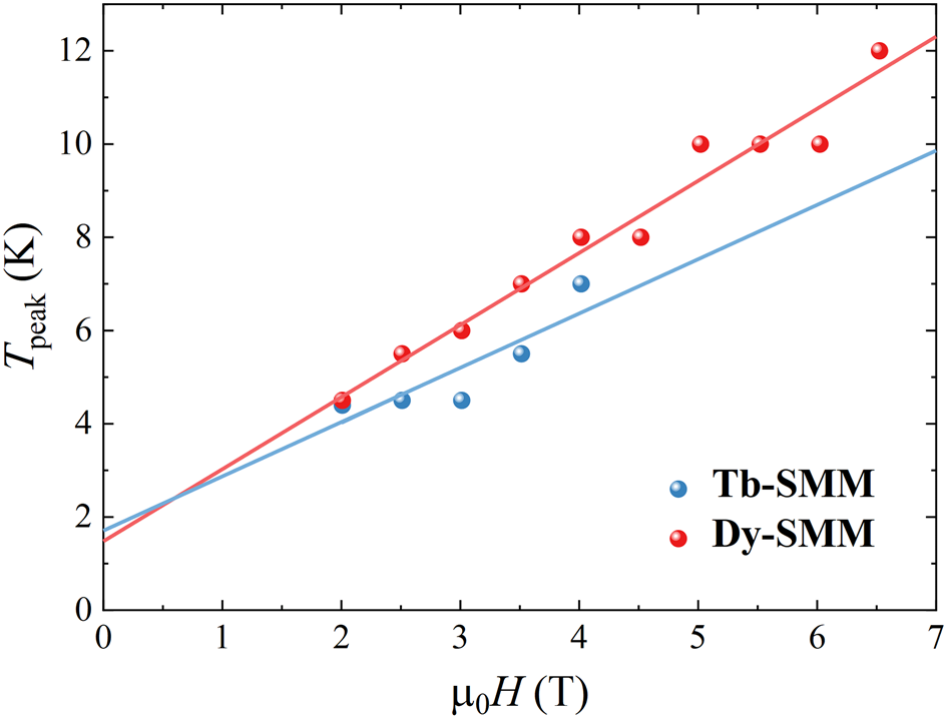
Field dependence of *T*_peak_ for **Tb-SMM** and **Dy-SMM**. The solid lines are the best fits to the linear function.

Figure 10 shows field dependence of temperature at which the − Δ*S*_R_ reveals a peak (*T*_peak_). The peaks could be observed only under certain conditions: *T* = 4.5–7 K and µ_0_*H* = 2–4 T for **Tb-SMM** and *T* = 4.5–16 K and µ_0_*H* = 2–7 T for **Dy-SMM**. For both compounds, the *T*_peak_ shifts to higher temperatures with increasing the magnetic field. Magnetic field splits the energy levels due to the Zeeman splitting. The higher the field, the greater the splitting. In higher fields, stronger thermal fluctuations are required to populate the shifted states. Therefore, the temperature of the *T*_peak_ is increasing with increasing magnetic field. The solid lines in Fig. 10 represent the best linear fit to the obtained points giving *a* = 1.24(40) K/T, *b* = 1.5(1.2) K and *a* = 1.55(10) K/T, *b* = 1.51(46) K for **Tb-SMM** and **Dy-SMM** respectively.

## Conclusions

The single crystal MCE of Dy^III^ and Tb^III^ based magnetic clusters were investigated in easy direction *c*∥*H* and hard plane *ab*∥*H*. It was shown that the presence of large magnetic anisotropy can have a substantial impact on the magnetic entropy change in two perpendicular orientations. The MCE for *c*∥*H* is higher in magnitude than for *ab*∥*H* and has a maximum peak, which is not the case for *ab*∥*H*. Because of these properties, the detailed research of RMCE was done for both studied compounds.

Although **Tb-SMM** reveals lower values of conventional MCE than **Dy-SMM**, regardless of temperature and field conditions, the efficiency of RMCE is greater for **Tb-SMM** due to substantially higher magnetic anisotropy of the Tb compound. The maximum of the entropy change − Δ*S*_max_ for RMCE was found at *T* = 2.0 K with − Δ*S*_max_ = 3.94 J K^−1^ kg^−1^ in µ_0_*H* = 1.3 T for **Tb-SMM** and − Δ*S*_max_ = 3.3 J K^−1^ kg^−1^ in µ_0_*H* = 1.1 T for **Dy-SMM**.

The performance of RMCE evaluated from *TEC* (*T*_lift_ = 5 K) is comparable with conventional MCE in all measured magnetic fields for **Tb-SMM** and up to approximately µ_0_*H* = 1 T for **Dy-SMM** (the difference between *TEC*s for RMCE and MCE was less than 10%). *TECs* obtained for **Tb-SMM** and **Dy-SMM** are almost the same up to µ_0_*H* = 1.5 T, but for higher magnetic fields, **Dy-SMM** outperforms **Tb-SMM** by 9% in µ_0_*H* = 4 T and 18% in µ_0_*H* = 7 T based on that figure of merit. However, *TEC* for RMCE indicates that the **Tb-SMM** single crystal is a better material for magnetocaloric cooling, with 12% higher *TEC* in µ_0_*H* = 1 T and 18% higher *TEC* in µ_0_*H* = 4 T than the corresponding *TEC* for **Dy-SMM**.

The relative efficiency of RMCE was calculated as the ratio RMCE/MCE_*c*∥*H*_ between entropy changes. The best efficiency is obtained at low magnetic fields, reaching almost 100% at all temperatures studied for **Tb-SMM** and 95% at *T* = 2.0 K for **Dy-SMM**. The peak position of the entropy change for RMCE moves towards higher temperatures with increasing magnitude of the magnetic field. The mutual relation between peak position coordinates (temperature, magnetic field) may be described using a linear function.

## Materials and methods

The single crystals of **Tb-SMM**^36^ and **Dy-SMM**^37^ were synthesized according to the literature procedures.

All the magnetic measurements were carried out with the MPMS XL magnetometer from Quantum Design. Single crystals of each compound were aligned in predetermined directions and mounted with Varnish GE adhesive. The plates with the single crystal were attached to a low-signal plastic straw to keep the specified orientation of the sample with respect to the magnetic field.

The studies were performed in two single crystal orientations: *ab*∥*H* and *c*∥*H*. The isothermal magnetization *M*(*H*) was collected at *T* = 2–80 K in the field range of µ_0_*H* = 0–7 T for **Tb-SMM** in the *c*∥*H* orientation and **Dy-SMM** for both geometries. The magnetic field range for **Tb-SMM** for *ab*∥*H* was reduced to µ_0_*H* = 0–4 T because the strong magnetic torque leads to breaking the sample in higher fields. The dc magnetic susceptibility *χ*(*T*) was measured during the cooling from *T* = 300 K to *T* = 2.0 K under µ_0_*H* = 0.1 T. The mass of single crystals was 6.51 mg for **Tb-SMM** and 1.87 mg for **Dy-SMM**. All measurements were corrected for diamagnetic contribution using Pascal’s constants^55^.

## Data Availability

The datasets used and/or analyzed during the current study are available from the corresponding author on reasonable request.
